# Associations Between Physical Activity and Cardiometabolic Risk Factors Assessed in a Southern California Health Care System, 2010–2012

**DOI:** 10.5888/pcd11.140196

**Published:** 2014-12-18

**Authors:** Deborah Rohm Young, Karen J. Coleman, Eunis Ngor, Kristi Reynolds, Margo Sidell, Robert E. Sallis

**Affiliations:** Author Affiliations: Karen J. Coleman, Eunis Ngor, Kristi Reynolds, Margo Sidell, Department of Research and Evaluation, Kaiser Permanente Southern California, Pasadena, California; Robert E. Sallis, Department of Family Medicine, Fontana Medical Center, Kaiser Permanente Southern California, Fontana, California.

## Abstract

**Introduction:**

Risk factors associated with many chronic diseases can be improved through regular physical activity. This study investigated whether cross-sectional associations between physical activity, assessed by the Exercise Vital Sign (EVS), and cardiometabolic risk factors can be detected in clinical settings.

**Methods:**

We used electronic records from Kaiser Permanente Southern California members (N = 622,897) to examine the association of EVS category with blood pressure, fasting glucose, random glucose, and glycosylated hemoglobin. Adults aged 18 years or older with at least 3 EVS measures between April 2010 and December 2012, without comorbid conditions, and not taking antihypertension or glucose-lowering medications were included. We compared consistently inactive (EVS = 0 min/wk for every measure) with consistently active (EVS ≥150 min/wk) and irregularly active (EVS 1–149 min/wk or not meeting the consistently active or inactive criteria) patients. Separate linear regression analyses were conducted controlling for age, sex, race/ethnicity, body mass index, and smoking status.

**Results:**

Consistently active women had lower systolic (−4.60 mm Hg; 95% confidence interval [CI], −4.70 to −4.44) and diastolic (−3.28 mm Hg; 95% CI, −3.40 to −3.17) blood pressure than inactive women. Active men had lower diastolic blood pressure than inactive men. Consistently active patients (women, −5.27 mg/dL [95% CI, −5.56 to −4.97]; men, −1.45 mg/dL [95% CI, −1.75 to −1.16] and irregularly active patients (women, −4.57 mg/dL [95% CI, −4.80 to −4.34]; men, −0.42 mg/dL [95% CI, −0.66 to −0.19]) had lower fasting glucose than consistently inactive patients. Consistently active and irregularly active men and women also had favorable random glucose and HbA1c compared with consistently inactive patients.

**Conclusion:**

Routine clinical physical activity assessment may give health care providers additional information about their patients’ cardiometabolic risk factors.

## Introduction

During the past 60 years, many studies have demonstrated that regular physical activity reduces illness and death from numerous diseases, including coronary heart disease (1,2), hypertension (3,4), diabetes (5,6), and stroke (7). Exercise training studies and more generalizable population-based physical activity interventions show that risk factors associated with these chronic diseases, including blood pressure (8,9), blood glucose (10,11), and glycemic control (10,12), can be improved. 

Most of the relevant literature includes adults who have been recruited into some type of study on the basis of strict selection criteria, limiting the generalizability of results to those responding to recruitment efforts (13). Few studies have examined associations between physical activity and cardiometabolic risk factors in community settings, such as health care organizations, findings of which may provide greater generalizability than those from the existing literature. The generalizability of health care settings will only increase as the Affordable Care Act is implemented and all Americans are required by law to obtain health care coverage. By studying health care settings, we have the unique opportunity to provide real-world guidance to health care providers and their patients about how health behaviors, such as physical activity, can provide direct benefits to patients.

In 2009, Kaiser Permanente Southern California (KPSC) created and implemented an Exercise Vital Sign (EVS) to be assessed, along with height, weight, and blood pressure, at every adult outpatient visit (14). This study used the EVS to determine whether associations between moderate to vigorous physical activity (MVPA) and blood pressure, blood glucose, and glycosylated hemoglobin (HbA1c) measures could be detected in clinical settings.

## Methods

### Setting

KPSC is an integrated health care system that serves approximately 3.6 million residents in Southern California at 14 medical centers and more than 200 medical offices. Racial/ethnic makeup, neighborhood education, and household income are generally similar to that of the area population, with marginal underrepresentation of people with very low income and those with high education (15). In addition to patients covered by employer health plans and other insurance options, KPSC members include patients from Medicare and Medicaid programs. The study was approved by the KPSC institutional review board.

### Participants

The study period was from April 1, 2010, through December 31, 2012. Inclusion criteria were adults aged 18 years or older as of April 1, 2010, who were health plan members during this time (with an allowable 2-month gap), and had at least 3 outpatient visits during the study period with an EVS measurement on each of these visits (Figure).

**Figure:**
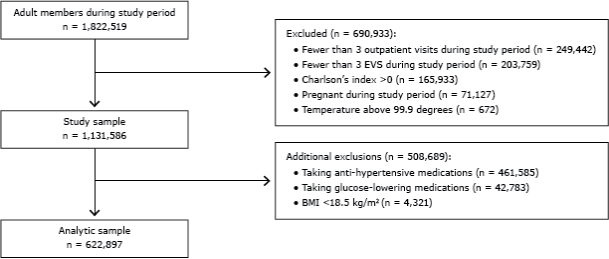
Study inclusion and exclusion criteria of Kaiser Permanente Southern California patients, 2010–2012. Abbreviations: BMI, body mass index; EVS, Exercise Vital Sign.

We also required that patients not have any major comorbidities and that all outcomes were abstracted from outpatient settings rather than hospitals or emergency departments. Physical activity and cardiometabolic risk factors can be altered by diseases or chronic conditions, which may mask true associations that we would not be able to detect. This requirement ensured that we would select a generally healthy population. Presence of comorbid conditions was assessed with a modified Charlson comorbidity index (16), which used diagnosis codes for 22 health conditions identified from 1 year before the study (April 1, 2009) through December 31, 2012, to create a summary score. We applied an additional inclusion criterion for the blood pressure analyses by requiring that each EVS measurement coincide with a blood pressure measure in the same setting.

Exclusion criteria were having Charlson’s comorbidity index greater than 0, being underweight (having a body mass index [BMI] <18.0 kg/m^2^), being pregnant during the study period, and having a body temperature above 99.9 degrees on a particular visit, which may indicate an illness and may affect both MVPA and blood pressure. In addition, patients could not be taking antihypertensive medications or glucose-controlling medications. We added these exclusions because the medications may mask possible effects from MVPA (Figure).

### Exercise Vital Sign

The EVS has been described in detail elsewhere (14). Briefly, trained medical assistants or nurses asked patients 2 questions at each outpatient visit: “On average, how many days per week do you engage in moderate to strenuous exercise (like a brisk walk)?” and “On average, how many minutes do you engage in exercise at this level?” Response choices for days were 0 to 7, and minutes were recorded as 0, 10, 20, 30, 40, 50, 60, 90, 120, and 150 minutes or more. The responses were recorded in each patient’s electronic medical record (EMR), and the associated software calculated minutes per week of MVPA. The EVS has good face and discriminant validity (14). Categories of *consistently physically active* (EVS ≥150 min/wk at all assessments during the study period), *consistently physically inactive* (EVS of 0 min/wk at all assessment periods), and *irregularly physically active* (EVS of 1–149 min/week, inconsistently active, or inconsistently inactive) were created for this study.

### Clinical measures

Blood pressure was also measured at every KPSC outpatient visit by trained medical assistants or nurses. After a 5-minute seated rest, blood pressure was measured using standard procedures with the arm supported at heart level. An automated sphygmomanometer, Welch Allyn Connex series (Welch Allyn, Inc), was used; if the measure was elevated (≥140/90 mm Hg), a second measurement was taken. Results were recorded in the EMR. All blood pressure measurements taken during the study period were averaged to create a mean blood pressure for each individual.

All laboratory tests and results were tracked through a laboratory management system, which was incorporated into the patient’s EMR. Because of varying clinical practices across the region, patients may have had a combination of fasting blood glucose, random blood glucose, and HbA1c in their records. All were measured from a blood draw from the antecubital region of the arm by a phlebotomist at a KPSC laboratory. The reference laboratories at KPSC were all Clinical Laboratory Improvement Amendment certified. All results during the study period were averaged for each patient and used for analysis.

### Covariates and analyses

Date of birth (to calculate age), race/ethnicity, and sex were obtained from electronic membership files. Smoking status was obtained from self-report during outpatient visits and recorded in the EMR. Height (m^2^) and weight (kg) were measured at each outpatient visit, values were recorded in the EMR, and BMI was calculated. All values obtained for each patient during the study period were averaged for the analyses.

Descriptive data were derived from frequency counts for categorical variables and means and standard deviations (SDs) for continuous variables. Separate multivariable linear regression models were conducted using systolic blood pressure, diastolic blood pressure, fasting glucose, random glucose, and HbA1c as the outcomes. Each analysis included the main predictor of physical activity status (consistently physically inactive, irregularly physically active, consistently physically active) and controlled for age, sex, race/ethnicity, BMI, and smoking status. The inactive group was the reference for the analyses. Analyses were also run with a sex*activity interaction variable because the descriptive data suggested differences in the exposure for some of the outcome variables. The interaction term was significant (*P* < .001), so results are presented stratified by sex. We calculated 95% confidence intervals (CIs) around the regression coefficients. Analyses were conducted using SAS Enterprise Guide 4.3 (SAS Institute Inc).

## Results

There were 622,897 patients (59% women) who met study criteria. Fasting glucose information was available for 401,635 patients, random glucose for 218,506, and HbA1c values for 158,653. Mean (SD) age was 44.2 (14.5) years (Table 1). Approximately one-third of patients were non-Hispanic white, and approximately one-third were Hispanic. On average, the sample was overweight (BMI = 28.0 [5.7]), and approximately 10% of the sample (more men than women) was classified as consistently physically active. Mean systolic and diastolic blood pressure, mean fasting glucose, random glucose, and HbA1c levels were lower for women than men. Adults who did not meet study criteria were more likely to be older (51.4 [17.6] years) and less likely to be female (50%) or Hispanic (27%). Smoking status was similar among patients who were included (12%) and excluded (11%) in the study sample.

**Table 1:** Sample Size and Sex-Specific Characteristics of Kaiser Permanente Southern California Patients, 2010–2012

Characteristic^a^	Overall (N = 622,897)	Women (n = 369,120)	Men (n = 253,777)
**Mean age, y (SD)**	44.2 (14.5)	44.7 (14.4)	43.5 (14.5)
**Race/ethnicity**			
Non-Hispanic white	133,370 (36.1)	92,670 (36.5)	226,040 (36.3)
Hispanic	220,677 (35.4)	134,755 (36.5)	85,922 (33.9)
Asian/Pacific Islander	57,338 (9.2)	36,787 (10.0)	20,551 (8.1)
Black	49,245 (7.9)	31,611 (8.6)	17,634 (6.9)
Unknown/other	69,596 (11.2)	32,597 (8.8)	36,999 (14.6)
**Mean body mass index, kg/m^2^ (SD)**	28.0 (5.7)	27.7 (6.1)	28.5 (5.1)
**Current smoker**	73,427 (11.8)	32,872 (8.9)	40,555 (16.0)
**Consistently physically inactive^b^**	52,904 (8.5)	30,002 (8.1)	22,902 (9.0)
**Irregularly physically active^c^**	506,936 (81.4)	308,594 (83.6)	198,342 (78.2)
**Consistently physically active^d^**	63,056 (10.1)	30,524 (8.3)	32,532 (12.8)
**Mean systolic blood pressure, mm Hg (SD)**	119.5 (10.3)	117.4 (10.6)	122.6 (9.0)
**Mean diastolic blood pressure, mm Hg (SD)**	71.9 (7.3)	70.7 (7.2)	73.7 (7.1)
**Mean fasting glucose, mg/dL (SD)**	95.0 (14.0)	93.2 (12.6)	97.6 (15.5)
**Mean random glucose, mg/dL (SD)**	101.6 (22.9)	100.0 (20.6)	104.1 (26.1)
**Mean HbA1c, % (SD)**	5.9 (0.7)	5.8 (0.6)	5.9 (0.7)

Abbreviations: SD, standard deviation; HbA1c, hemoglobin A1c.

a Values presented as no. (%), unless otherwise indicated.

b Consistently physically inactive defined as 0 weekly minutes of moderate to vigorous physical activity consistently during the study period.

c Irregularly physically active defined as from 1 to 149 weekly minutes of moderate to vigorous physical activity or inconsistently inactive or active during the study period.

d Consistently physically active defined as ≥150 weekly minutes of moderate to vigorous physical activity consistently during the study period.

### Blood pressure

Consistently physically active women had lower systolic (−4.60 mm Hg; 95% CI, −4.70 to −4.44) and diastolic (−3.28 mm Hg; 95% CI, −3.40 to −3.17) blood pressure than did inactive patients (Table 2). Irregularly physically active women also had lower systolic (−4.85 mm Hg; 95% CI, −4.97 to −4.73) and diastolic (−3.40 mm Hg; 95% CI, −3.49 to −3.31) blood pressure than did inactive patients. Compared with consistently inactive men, irregularly active men had slightly but significantly lower systolic blood pressure, and consistently physically active men had a slightly higher systolic blood pressure. Consistently physically active men (−1.79 mm Hg; 95% CI, −1.90 to −1.68) and irregularly active men (−0.78 mm Hg; 95% CI, −0.88 to −0.69) had lower diastolic blood pressure than did consistently inactive men. Physical activity–sex interactions were significant (*P* < .001), and results indicated a greater magnitude of difference in systolic and diastolic blood pressure for consistently physically active women than for consistently physically active men and for irregularly active women than for irregularly active men.

**Table 2:** Associations Between Physical Activity and Cardiometabolic Risk Factors,^a^ Kaiser Permanente Southern California Patients, 2010–2012

Parameter/Physical Activity Level^b^	Women^c^	Men^c^
	Regression Coefficient (95% CI)	
**Systolic blood pressure, mm Hg (n = 662,639)**		
Irregularly physically active	−4.85 (−4.97 to −4.73)	−0.09 (−0.22 to −0.03)
Consistently physically active	−4.60 (−4.70 to −4.44)	0.98 (0.83 to 1.14)
**Diastolic blood pressure, mm Hg (n = 662,639)**		
Irregularly physically active	−3.40 (−3.49 to −3.31)	−0.78 (−0.88 to −0.69)
Consistently physically active	−3.28 (−3.40 to −3.17)	−1.79 (−1.90 to −1.68)
**Fasting glucose, mg/dL (n = 401,635)**		
Irregularly physically active	−4.57 (−4.80 to −4.34)	−0.42 (−0.66 to −0.19)
Consistently physically active	−5.27 (−5.56 to −4.97)	−1.45 (−1.75 to −1.16)
**Random glucose, mg/dL (n = 218,506)**		
Irregularly physically active	−5.33 (−5.86 to −4.80)	−1.17 (−1.71 to −0.63)
Consistently physically active	−7.59 (−8.30 to −6.88)	−4.10 (−4.80 to −3.39)
**HbA1c, % (n = 158,653)**		
Irregularly physically active	−0.12 (−0.14 to −0.11)	−0.05 (−0.07 to −0.03)
Consistently physically active	−0.15 (−0.17 to −0.13)	−0.13 (−0.15 to −0.11)

Abbreviations: CI, confidence interval; HbA1c, hemoglobin A1c.

a Physical activity measured by the Exercise Vital Sign measure (14); comparison group was consistently physically inactive (0 min/wk of moderate to vigorous physical activity during the study period).

b Irregularly physically active defined as from 1 to 149 weekly minutes of moderate to vigorous physical activity or inconsistently inactive or active during the study period; and consistently physically active defined as ≥150 weekly minutes of moderate to vigorous physical activity consistently during the study period.

c Analyses controlled for age, sex, race/ethnicity, body mass index, and smoking status, and include a sex*activity interaction term.

### Glucose and HbA1c

Fasting glucose was lower for consistently physically active women (−5.27 mg/dL; 95% CI, −5.56 to −4.97) and irregularly active women (−4.57 mg/dL; 95% CI, −4.80 to −4.34) than that for consistently inactive women. The same was true for men; consistently active men (−1.45 mg/dL; 95% CI, −1.75 to −1.16) and irregularly active men (−0.42 mg/dL, 95% CI, −0.66 to −0.19) had lower fasting glucose than did those who were consistently inactive (Table 2). Similar results were noted for random glucose and HbA1c. Physical activity/sex interactions were significant (*P* < .001). For all 3 glucose measures, consistently physically active women had a greater magnitude of difference than did the consistently active men (fasting glucose, −5.27 mg/dL vs −1.45 mg/dL; random glucose, −7.59 mg/dL vs −4.10 mg/dL; HbA1c, −0.15% vs −0.13%). Similarly, the magnitude of difference for the irregularly physically active women was greater than the irregularly active men for fasting glucose, random glucose, and HbA1c.

## Discussion

Our results demonstrate that, using the EVS as a measure of MVPA, generally healthy, consistently active, and irregularly physically active patients of both sexes have lower diastolic blood pressure, glucose, and HbA1c levels than patients who are consistently physically inactive. Consistently active and irregularly active women also have lower systolic blood pressure. For glucose and HbA1c, the most favorable values were for consistently physically active patients. Consistently active and irregularly active women had a greater magnitude of difference for all the cardiometabolic variables compared with similarly active men.

The associations we observed were modest and cross-sectional. However, even a small improvement in cardiometabolic risk factors has a profound impact on population health. Cook et al estimated that a 2 mm Hg reduction in diastolic blood pressure would decrease the US prevalence of hypertension by 17% and reduce the risk of coronary heart disease and stroke by 6% and 15%, respectively (17). Others have made similar conclusions (18,19). This amount of blood pressure reduction is slightly smaller than the differences we found between the physically active and the consistently inactive women. However, we did not observe the same results for men, which we are not able to explain. Other research results have found similar cross-sectional physical activity and blood pressure associations for men and women (20).

The difference in fasting glucose (approximately 3%) we found between the consistently physically active and consistently inactive patients is comparable to findings from lifestyle interventions in populations at risk for future cardiometabolic disease. Randomized trials of weight loss, dietary patterns, and physical activity interventions reported reductions of a similar magnitude for intervention compared with control group participants (21–23). For example, the Diabetes Prevention Program trial showed that the changes in fasting glucose and HbA1c levels from the lifestyle intervention were −4 mg/dL and −0.14%, respectively, over 1 year (21), similar to the difference we found between the active and inactive patients. This amount of change, along with other benefits of the lifestyle intervention, reduced the incidence of diabetes by 58% (21).

The random and fasting glucose and HbA1c results were graded, with favorable values among patients who were irregularly physically active compared with those who were consistently inactive and the most favorable values among patients who were consistently physically active. These results were not noted for blood pressure in which irregularly active women had blood pressure reductions similar to those of consistently active women. Although the exact amount of physical activity needed to produce blood pressure reductions has not been identified, it is recognized that a low amount of physical activity can improve blood pressure (24). In contrast, cross-sectional and intervention studies indicate that those in higher physical activity categories have lower glucose, lower HbA1c levels, or both, in a graded pattern (12,25). Different biological mechanisms are thought to be responsible for the blood pressure–lowering and glucose-lowering effects from physical activity (26), so these results are not unexpected.

We cannot explain why the magnitude of differences between the consistently active and consistently inactive patients was greater for women than for men. Other studies reported stronger correlations between physical activity and 2-hour glucose levels in men compared with women (Pearson correlations, −0.22 vs −0.11, respectively) (27) and increased odds of high plasma glucose levels at lower physical activity categories for men but not women (28). However, Ekelund et al did not find sex differences in change in physical activity and glucose levels over a 5.6-year follow-up period (29). Although there are no known physiological differences between men and women to explain our results, future studies should present sex-stratified results to determine whether there are differences in response to physical activity.

Our study has many limitations. It was cross-sectional, which limits the ability of making causal inferences. However, a multitude of investigations have demonstrated the effects of physical activity on blood pressure and glucose levels. The significant effects may not be clinically meaningful, given our very large sample size. The study population had to have had at least 3 outpatient visits during a 2.5-year period, which may make them substantively different from other generally healthy patients who have lower health care use. There are limitations with self-reported physical activity that have been noted elsewhere (30). In particular, the EVS refers to “exercise,” which implies leisure physical activity but not transportation or occupational activity that is captured in objective measures, such as accelerometers, or more comprehensive self-report instruments. However, the EVS provides similar estimates of physical activity as other population-based self-report surveys (14). The cross-sectional study was over a 33-month period during which there may have been changes in health status or health behaviors that we could not discern.

In conclusion, consistently physically active and irregularly active patients, as assessed by the EVS, have lower diastolic blood pressure, glucose, and HbA1c levels than patients who are consistently physically inactive. On a population level, the associations we observed were comparable with those needed to reduce the risk of coronary heart disease, stroke, and diabetes. If health care providers would routinely assess the physical activity of their patients and refer those who are physically inactive to effective physical activity programs, it may reduce the burden of future chronic diseases.
